# Prevalence and Associated Factors of Secondhand Smoke Exposure among Internal Chinese Migrant Women of Reproductive Age: Evidence from China’s Labor-Force Dynamic Survey

**DOI:** 10.3390/ijerph13040371

**Published:** 2016-04-01

**Authors:** Xiao Gong, Xiaofeng Luo, Li Ling

**Affiliations:** 1 Faculty of Medical Statistics and Epidemiology, School of Public Health, Sun Yat-sen University, Guangzhou 510080, China; x.gong@foxmail.com (X.G.); luoxiaof@lzu.edu.cn (X.L.); 2 Sun Yat-sen Center for Migrant Health Policy, Sun Yat-sen University, Guangzhou 510080, China

**Keywords:** internal migration, secondhand smoke, women of reproductive age, China

## Abstract

Secondhand smoke (SHS) is a major risk factor for poor health outcomes among women in China, where proportionately few women smoke. This is especially the case as it pertains to women’s reproductive health, specifically migrant women who are exposed to SHS more than the population at large. There are several factors which may increase migrant women’s risk of SHS exposure. This paper aims to investigate the prevalence and associated factors of SHS exposure among internal Chinese migrant women of reproductive age. The data used were derived from the 2014 Chinese Labor Dynamic Survey, a national representative panel survey. The age-adjusted rate of SHS exposure of women of reproductive age with migration experience was of 43.46% (95% CI: 40.73%–46.40%), higher than those without migration experience (35.28% (95% CI: 33.66%–36.97%)). Multivariate analysis showed that participants with a marital status of “Widowed” had statistically lower exposure rates, while those with a status of “Cohabitation” had statistically higher exposure. Those with an undergraduate degree or above had statistically lower SHS exposure. Those with increasing levels of social support, and those who currently smoke or drink alcohol, had statistically higher SHS exposure. Participants’ different work-places had an effect on their SHS exposure, with outdoor workers statistically more exposed. Our findings suggest that urgent tobacco control measures should be taken to reduce smoking prevalence and SHS exposure. Specific attention should be paid to protecting migrant women of reproductive age from SHS.

## 1. Introduction

Tobacco use is a worldwide public health problem, and is a major preventable risk factor of diseases and death [1]. Estimated total tobacco-attributable deaths will reach 8.3 million in 2030, mainly from low- and middle-income countries [2]. Secondhand smoke (SHS) is almost as risky as smoking itself. SHS not only impairs the health of those exposed [3,4,5], but also leads to adverse reproductive outcomes for exposed reproductive women, such as infertility [6], spontaneous abortion [7], low birthweight [8], preterm birth [9,10,11], infant death [12], and is also dangerous to the health of children [13,14].

China has a high tobacco prevalence and high levels of secondhand smoke exposure, which results in a particularly high smoking-related disease burden that is still on the rise [15,16]. As reported by the Global Adult Tobacco Survey (GATS) [17], in 2010, an estimated 28.1% of adults in China (52.9% of men and 2.4% of women) were smokers. Women, although with a relatively low smoking rate, are exposed to tremendous hazards of the high tobacco prevalence, bearing nearly 80% of the total disease burden of SHS [18]. As reported by nonsmokers, 72.4% were exposed to secondhand smoke with 38.0% exposed on a daily basis [19]. However, most Chinese women do not fully recognize these hazards and therefore do not successfully avoid SHS exposure [17], even before and/or during their pregnancy [20].

The Chinese population has experienced a massive internal migration tide since the economic reform and opening-up. The number of migrants leaving their officially registered places of residence has been estimated as 50 million in 1990, 121 million in 2000, and 253 million in 2014 [21]. Of these 253 million 78% are between the ages of 15 to 59, and half of them are women [21]. Due to the Chinese *hukou* (household registry) system, these migrants do not have the same rights as the local permanent residents. These rights include benefits such as social security and welfare, which are tied to permanent residency registration. In addition to those institutional reasons, leaving their previous residences and living a migratory life, is in itself a hazardous situation for those migrants. All of the above reasons make migrants a particularly vulnerable population.

According to a series of studies of different groups of migrants, migrants have higher smoking rates than the general population, ranging from 48.7% to 64.9% for males, and from 2.1% to 10.9% for females [22,23,24,25,26]. Although we believe migrants have a high tobacco contact rate, studies aiming to investigate the SHS exposure of the migrant population, especially migrant reproductive women are still lacking. Some characteristics of reproductive migrant women, such as younger average age, low education levels, and migratory lifestyles as well as their work and living environments [5,21,27] may increase their exposure to SHS. Several studies have reported that SHS exposure among migrant women is between 62% and 64% [28,29]. These estimates were based on subgroups of migrants (including several typical employed populations) and are not representative of the entire migrant population. Additionally, the definition of SHS exposure in those studies did not consider the exposure time duration, but only tested whether there was any chance of exposure to SHS. This loose definition weakens the explanatory power of these studies on the health impact estimate of SHS exposure.

Our study uses a nationally representative sample to estimate the SHS exposure (defined as more than 15 min daily exposure) rate among internal Chinese migrant women of reproductive age. It also makes an age-adjusted rates comparison to those without migration experience, and investigates associated factors which may impact the exposure.

## 2. Methods

### 2.1. Data and Sampling

The data were derived from the 2014 Chinese Labor Dynamic Survey (CLDS), which was the second wave of a nationally representative panel survey [30]. The survey was sponsored by the Center for Social Survey at Sen Yat-sen University. It used computer assisted personal interviewing methodology and sampled with a multi-stage stratified sampling method. The survey targeted individuals age 15 to 65, and covered 29 out of 31 province-level administrative areas of mainland China (excluding Tibet and Hainan). The second wave was conducted in the same communities as the first wave (applied in year 2012). Of a total of 23,594 respondents in the second wave, 10,053 were follow up samples from the first wave, and 13,541 were newly added participants. We extracted and analyzed the data from women aged 15 to 49, the range usually used as reproductive age in World Health Organization (WHO) documents [31].

### 2.2. Variable Definitions

#### 2.2.1. Secondhand Smoke

SHS exposure has been defined as exposure to another person’s tobacco smoke for at least 15 min per day for more than one day every week [32]. Participants were asked “the average frequency they were exposed to another person’s tobacco smoke for at least 15 min per day”, with choices of “Almost every day”, “Equal to or more than 3 days per week”, “Equal to or more than 1 day and less than 3 days per week”, “Less than 1 day per week” and “No exposure”. Participants who chose one of the first three choices were recognized as SHS exposed.

#### 2.2.2. Migration Experience

In this study, we categorize the overall population into two groups, those with migration experience and those without migration experience. We define people with migration experience as those who had the experience of leaving their registered residence (according to the *hukou* system) for more than 6 months. This “6-months” definition has been widely used by the Chinese government as a handy management rule. In the Chinese academic community, researchers have also accepted this definition as the migratory lifestyle may require a period to affect the behavior or health of those migrants.

#### 2.2.3. Demographic Variates

Demographic characteristics used in this paper include age group (“15–19”, “20–29”, ”30–39”, ”40–49”), marital status (“Single”, “Married: First Marriage”, “Married: Non-first Marriage”, “Divorced” and “Widowed”), and education level (“Primary School or Illiteracy”, “Secondary School”, “High School or Equivalent” and “Undergraduate or Above”).

#### 2.2.4. Other Covariates

The social support variable was first a score of the linear combination of three questions examining the numbers of people that the participant could refer to when they needed to speak their minds, discuss important issues or borrow money equivalent to 5000 RMB (approximately 800 US dollars). Then we divided it into four levels according to the score’s distribution characteristics, which were named “No Support”, “Low Support”, “Moderate Support”, and “High Support”. Self-reported health status was a Likert 5-scale item, with “Excellent”, “Good”, “Fair”, “Poor” and “Very Poor” as the five scale answers. Tobacco smoke and alcohol use status were also recorded by the CLDS. Participants were categorized into “Smokers” and “Non-smokers” according to whether they had smoked in the past 30 days. Since women generally drink less alcohol in China, we categorized them into two levels according to the data distribution: “Less than once a week” and “Once a week or more”. Work place information was also included in our study, and recoded as “Outdoors”, “Factory Workshop”, “Indoor Business Places”, “Office”, “Home (working from home)” and “Unfixed or Other (including those workers with unfixed workplaces and those without jobs)”.

### 2.3. Statistical Analysis

The statistical software R (Version 3.2.0. R Foundation for Statistical Computing, Vienna, Austria) was used for statistical analysis. To make better comparisons of SHS exposure between different groups with different age structures, standardized rates were calculated, and a direct method was used [33,34]. Univariate and multivariate logistic regressions were performed to identify the factors associated with SHS exposure. *p*-values of less than 0.05 were considered statistically significant.

## 3. Results

### 3.1. Demographics of the Study Population

We had 7617 women of reproductive age identified out of all survey participants (Table 1). Of those participants, 41.1% were between 40 and 49; the majority were married and on their first marriage (76.6%). Most of the participants were not well educated (29.7% were primary school or below, and 34.4% were secondary school).

### 3.2. SHS Exposure Status by Migration Experience

Of the 7617 participants, 2492 had migration experience and 5125 did not. Among participants with migration experience, 1094 (43.9%) had been exposed to SHS. 1806 (35.2%) of those without migration experience had been exposed (Table 1).

**Figure 1:**
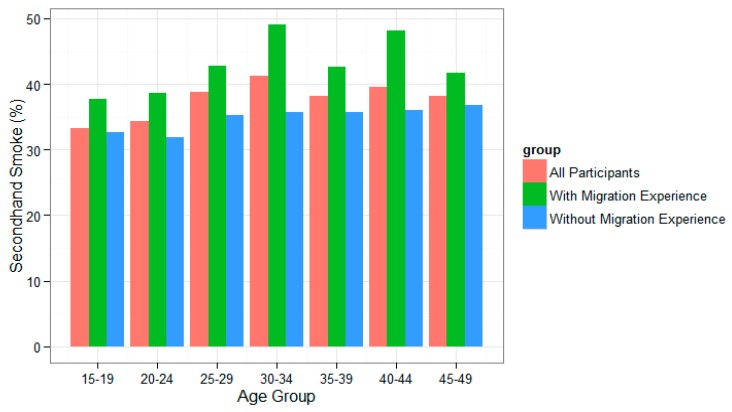
Secondhand smoking rates of different age groups for all participants.

Figure 1 demonstrates the SHS exposure rates of different age groups (“15–19”, “20–24”, “25–29”, “30–34”, “35–39”, “40–44”, “45–49”) for all participants, participants with and participants without migration experience. It shows that SHS exposure rates among women with migration experience were higher than among those without migration experience.

SHS exposure rates also showed disparities between different age groups (Figure 1). The migrant population has an age structure different from the non-migrant population; migrants, on average, are younger [21]. Since age may be a confounding factor for the SHS exposure rates estimation and comparison, we calculated both the crude and the age-adjusted rates, as well as a 95% confidence interval.

For individuals with migration experience, the crude SHS exposure rate was 43.90%, with an age-adjusted rate of 43.46% (95% CI: 40.73%–46.40%). For individuals without migration experience, the crude SHS exposure rate was 35.24%, with an age-adjusted rate of 35.28% (95% CI: 33.66%–36.97%). 

### 3.3. Associated Factors of SHS Exposure: Results of Univariate and Multivariate Logistic Regressions

We tested the associated factors of SHS exposure among migrant women of reproductive age through univariate and multivariate logistic regression (Table 2). 

In the multivariate adjusted model, age group showed no significant difference. The participants with a marital status of “Widowed” had lower rates of exposure, while those with a status of “Cohabitation” had higher rates. Married participants had higher rates of exposure in the univariate model, but the effect disappeared after multivariate adjustment. Those with an undergraduate degree or above had lower SHS exposure rates. Those with low social support or above, and those who drank alcohol once a week or more, had higher SHS exposure rates. Participants’ different work places had an important effect on their SHS exposure. “Indoor Business Places”, “Office”, “Home” and “Unfixed or Other” had lower exposures than “Outdoors”.

## 4. Discussion

This paper estimates secondhand smoke exposure among migrant women of reproductive age, and compares their exposure rates with that of their non-migrant counterparts using a population representative sample. For the control group—participants without migration experience—the age-adjusted SHS exposure was 35.28% (95% CI: 33.66%–36.97%). These values are lower when compared to the rates from the 2010 China Adult Tobacco Survey (71.6%) [19], and from a study conducted on Jilin Province, China (60.6%) [35]. Other than the different age intervals considered, the most probable reason for this discrepancy is the variation in SHS definitions used. In our study, SHS required that the length of smoke exposure exceed a threshold of 15 min per day. Previous studies have not used this threshold, instead allowing for the usage of any length of exposure. Those participants who had experienced the migration process had a higher exposure to SHS. The age-adjusted exposure rate was 43.46% (95% CI: 40.73%–46.40%), more than 8% higher than participants without migration experience. This rate was also lower than the estimates in other migrants’ studies (62.0% of women 18 and above [28]; and 64.0% of women among 18–59 [29]). A possible explanation for the discrepancy may be the definition of SHS exposure mentioned above as well as specific sampling frameworks. Previous studies have sampled participants based on several typical occupations that may not be representative of the migrant as a whole. This suggests that the migration experience may be a factor that increases the chances of coming into contact with SHS.

In the analysis of associated factors of SHS exposure among migrant reproductive women, age group showed no significant results. Thus, we believe this implies that smokers, when they smoke, have no consciousness of protecting young women from SHS exposure. On the other hand, this may be related to the higher smoking rates among their peers of similar age [23,26]. Migrants tend to be younger than non-migrants [21]. High rates of exposure for young migrant women’s high SHS exposure rate may yield worse health outcomes for China as a whole. This requires urgent attention.

Marital status showed that the widowed had lower exposure rates, and those who cohabitated had higher exposure rates. The higher rates of those who were married was significant in univariate analysis, but insignificant in multivariate analysis. This may be because unmarried migrants have a higher education level and the difference between married and unmarried in the univariate model was explained away by education level. This may suggest that the smoking status of family members (husbands or people in a relationship) can be important in women’s SHS exposure [35]. Since almost all smokers are male (52.9% of men and 2.4% of women) in China, widows have less probability of being exposed to SHS.

Analysis of education level showed that the highly educated experience less SHS. This may be because the people around them are usually also highly educated as well and are therefore less likely to smoke [23,36]. On the other hand, the highly educated may tend to intentionally avoid SHS in their daily life, as they may have more knowledge of the hazards of SHS. Since migrants are generally at low education levels [21], the SHS problem of this population needs further attention.

Social support is a significant risk factor for SHS exposure. Participants with low to high social support have higher exposure than those without support. In China, tobacco smoking has strong socialization attributes and is often regarded as a social communication tool. Smokers tend to offer cigarettes to other people when they meet as a manner of showing respect. Many smokers even start conversations with this kind of respective cigarette offer. Furthermore, there is a tendency of persuading acquaintances to smoke (known as the contagious effect in literature [37,38,39]). In this social context that tolerates smoking, smoking naturally becomes an easy and effective way for migrants to develop social connections in a new environment. Although women are generally expected not to smoke in Chinese culture, they cannot avoid SHS in such social activities. The more social ties people have, the higher the probability they will be exposed to SHS.

Self-reported health status showed no statistically significant results in the model. However, we noticed an increase in the ORs as health degraded. We hypothesize that participants with poorer health status had work or lifestyles that were connected with smoking environments. This require further studies with larger sample sizes and more detailed questionnaire tools.

Smokers had higher SHS exposure rates than non-smokers. We speculate that smokers tend to smoke with other smokers, or go to smoking areas more frequently than non-smokers. Thus, smokers are more likely to be exposed to SHS.

Alcohol use is statistically related to SHS exposure in the models, and this is supported by other studies [40]. This is also consistent with Chinese cultural tendencies. Cigarettes and alcohol are often consumed simultaneously, and women who drink cannot avoid SHS while drinking.

Workplace analysis showed that indoor work is a significant protective factor for avoiding SHS exposure. Different venues have different SHS exposure rates [28,41]. Migrants usually work outdoors or in other places of high smoking rates where local residents have less interest [27]. Therefore, they are more likely to be exposed to SHS in their workplaces.

Considering the higher SHS exposure rates and the associated factors above, we believe that SHS is a serious problem for migrant reproductive women. We suggest that related policies or interventions be undertaken urgently. Firstly, migrant women should be made aware of the hazards of SHS through various health education programs. Secondly, smoke-free homes may be a good intervention choice since those married or cohabitating have higher rates of SHS exposure. Thirdly, culturally speaking, smoking should be recognized as a bad habit rather than a decent behavior. Smoking in front of others should be thought as shameful and should not be allowed. Additionally, tobacco related policies should be integrated into the occupational health system to reduce SHS exposure in the workplace. The high exposure rate is associated not only with the specific characteristics of those women, but also with the high smoking prevalence of the whole migrant population, as well as the greater population as a whole. To control SHS exposure, the government should take measures at once to implement smoke-free policies, and strengthen health education about the hazards of SHS for the society as a whole.

This study has several strengths and limitations. First, we explored the SHS exposure status of migrant reproductive women with SHS exposure defined as more than 15 min exposure, a metric which had not previously well studied in this population. Second, the data were derived from a national presentative survey. Thus, the prevalence estimation can be extrapolated to the larger population. With the comparison of non-migrants, the disadvantage of migrant reproductive women on SHS exposure is clearer. However, SHS exposure was assessed through self-reporting. Despite some recall bias, this method is still thought to be reliable. Moreover, we did not consider third-hand smoke (THS) in our paper. On the hazards of THS [42], we suggest that further studies use the definition of environmental tobacco smoke, which contains both SHS and THS [43,44]. Also, this large study is neither specific nor limited to tobacco exposure. Therefore, the factors associated with SHS exposure considered in this paper are limited. Several important variables, such as venues or sources of migrant women’s SHS exposure, should be studied in further research. Well studied places of SHS exposure would suggest specific smoke-free locations as policy priorities. Variables associated with migration experience, cannot be exactly extracted from CLDS. Factors such as length of migration time could affect the exposure status or associated factors. This also deserves further study.

## 5. Conclusions

SHS exposure among reproductive women with migration experience was higher than those without migration experience. Marital status, lower education level, higher social support, as well as more alcohol use and an outdoor workplace are associated factors of higher SHS exposure. Urgent tobacco control measures should be taken to reduce the smoking prevalence and SHS exposure rates. Specific attention should be addressed to protecting migrant women of reproductive age.

## Figures and Tables

**Table 1:** Demographic characteristics of participants by migration experience and secondhand smoke status.

Variables	Number	Participants With Migration Experience		Participants Without Migration Experience	
		SHS (N, %)	Non-SHS (N, %)	SHS (N, %)	Non-SHS (N, %)
Sample Size	7617	1094	1398	1806	3319
Age Group					
15–19	732 (9.6)	29 (2.7)	48 (3.4)	214 (11.8)	441 (13.3)
20–29	1754 (23.0)	308 (28.2)	438 (31.3)	340 (18.8)	668 (20.1)
30–39	1998 (26.2)	355 (32.4)	418 (29.9)	437 (24.2)	788 (23.7)
40–49	3133 (41.1)	402 (36.7)	494 (35.3)	815 (45.1)	1422 (42.8)
Marital Status					
Single	1425 (18.7)	99 (9.0)	185 (13.2)	362 (20.1)	779 (23.5)
Married: First Marriage	5833 (76.6)	944 (86.3)	1146 (82)	1380 (76.5)	2363 (71.2)
Married: Non-first Marriage	141 (1.9)	23 (2.1)	21 (1.5)	25 (1.4)	72 (2.2)
Divorced	82 (1.1)	9 (0.8)	17 (1.2)	14 (0.8)	42 (1.3)
Widowed	62 (0.8)	4 (0.4)	21 (1.5)	10 (0.6)	27 (0.8)
Cohabitation	72 (0.9)	15 (1.4)	8 (0.6)	14 (0.8)	35 (1.1)
Education Level					
Primary School or Below	2263 (29.7)	325 (29.7)	366 (26.2)	568 (31.5)	1004 (30.3)
Secondary School	2618 (34.4)	429 (39.2)	493 (35.3)	611 (33.8)	1085 (32.7)
High School or Equivalent	1355 (17.8)	177 (16.2)	229 (16.4)	331 (18.3)	618 (18.6)
Undergraduate or Above	1381 (18.1)	163 (14.9)	310 (22.2)	296 (16.4)	612 (18.4)
Social Support					
No Support	1022 (13.4)	144 (13.2)	242 (17.3)	208 (11.5)	428 (12.9)
Low Support	1508 (19.8)	256 (23.4)	330 (23.6)	346 (19.2)	576 (17.4)
Moderate Support	2789 (36.6)	403 (36.8)	479 (34.3)	694 (38.4)	1213 (36.5)
High Support	2298 (30.2)	291 (26.6)	347 (24.8)	558 (30.9)	1102 (33.2)
Self-reported Health Status					
Excellent	1925 (25.3)	231 (21.1)	322 (23)	429 (23.8)	943 (28.4)
Good	3328 (43.7)	462 (42.2)	600 (42.9)	792 (43.9)	1474 (44.4)
Fair	1750 (23)	303 (27.7)	379 (27.1)	397 (22)	671 (20.2)
Poor	540 (7.1)	87 (8.0)	88 (6.3)	169 (9.4)	196 (5.9)
Very Poor	74 (1.0)	11 (1.0)	9 (0.6)	19 (1.1)	35 (1.1)
Tobacco Use					
Non-smokers	7524 (98.8)	1072 (98.0)	1390 (99.4)	1767 (97.8)	3295 (99.3)
Smokers	93 (1.2)	22 (2.0)	8 (0.6)	39 (2.2)	24 (0.7)
Alcohol Use					
Less than once a week	7437 (97.6)	1052 (96.2)	1373 (98.2)	1747 (96.7)	3265 (98.4)
Once a week or more	180 (2.4)	42 (3.8)	25 (1.8)	59 (3.3)	54 (1.6)
Work Place					
Outdoors	1723 (22.6)	236 (21.6)	213 (15.2)	473 (26.2)	801 (24.1)
Factory Workshop	690 (9.1)	156 (14.3)	186 (13.3)	138 (7.6)	210 (6.3)
Indoor Business Places	1020 (13.4)	194 (17.7)	272 (19.5)	206 (11.4)	348 (10.5)
Office	1190 (15.6)	188 (17.2)	271 (19.4)	293 (16.2)	438 (13.2)
Home	294 (3.9)	42 (3.8)	71 (5.1)	71 (3.9)	110 (3.3)
Unfixed or Other	2700 (35.4)	278 (25.4)	385 (27.5)	625 (34.6)	1412 (42.5)

**Table 2:** Associated factors of secondhand smoke among migrant women of reproductive age through univariate and multivariate logistic regression (*N* = 2492).

Variables	Univariate Logistic Regression		Multivariate Logistic Regression	
	OR (95% CI)	*p*–Value	OR (95% CI)	*p*–Value
Age Group				
15–19	Reference		Reference	
20–29	1.16 (0.72–1.91)	0.538	1.18 (0.69–2.04)	0.554
30–39	1.41 (0.87–2.30)	0.166	1.21 (0.68–2.18)	0.512
40–49	1.35 (0.84–2.20)	0.223	1.07 (0.60–1.93)	0.827
Marital Status				
Single	Reference		Reference	
Married: First Marriage	1.54 (1.19–2.00)	0.001	1.24 (0.89–1.73)	0.207
Married: Non–first Marriage	2.05 (1.08–3.91)	0.028	1.46 (0.72–2.95)	0.291
Divorced	0.99 (0.41–2.25)	0.98	0.94 (0.37–2.25)	0.896
Widowed	0.36 (0.10–0.97)	0.065	0.21 (0.06–0.62)	0.009
Cohabitation	3.50 (1.47–8.97)	0.006	3.01 (1.24–7.86)	0.018
Education Level				
Primary School or Below	Reference		Reference	
Secondary School	0.98 (0.80–1.19)	0.841	1.02 (0.82–1.27)	0.853
High School or Equivalent	0.87 (0.68–1.11)	0.27	0.94 (0.71–1.24)	0.653
Undergraduate or Above	0.59 (0.46–0.75)	<0.001	0.58 (0.42–0.79)	0.001
Social Support				
None Support	Reference		Reference	
Low Support	1.30 (1.00–1.70)	0.048	1.34 (1.03–1.76)	0.032
Moderate Support	1.41 (1.11–1.81)	0.006	1.51 (1.18–1.95)	0.001
High Support	1.41 (1.09–1.83)	0.009	1.59 (1.22–2.08)	0.001
Self–reported Health Status				
Excellent	Reference		Reference	
Good	1.07 (0.87–1.32)	0.505	1.06 (0.85–1.31)	0.607
Fair	1.11 (0.89–1.40)	0.349	1.00 (0.79–1.27)	0.987
Poor	1.38 (0.98–1.94)	0.065	1.28 (0.89–1.84)	0.188
Very Poor	1.70 (0.69–4.29)	0.244	1.73 (0.67–4.59)	0.255
Tobacco Use				
Non–smokers	Reference		Reference	
Smokers	3.57 (1.65–8.56)	0.002	3.50 (1.55–8.78)	0.004
Alcohol Use				
Less than once a week	Reference		Reference	
Once a week or more	2.19 (1.34–3.67)	0.002	2.24 (1.34–3.81)	0.002
Work Place				
Outdoors	Reference		Reference	
Factory Workshop	0.76 (0.57–1.00)	0.053	0.77 (0.57–1.03)	0.074
Indoor Business Places	0.64 (0.50–0.84)	0.001	0.68 (0.51–0.90)	0.008
Office	0.63 (0.48–0.81)	<0.001	0.86 (0.62–1.18)	0.343
Home	0.53 (0.35–0.81)	0.004	0.55 (0.35–0.85)	0.007
Unfixed or Other	0.65 (0.51–0.83)	<0.001	0.69 (0.53–0.89)	0.004

## References

[1] World Health Organization (WHO) (2011). WHO Report on the Global Tobacco Epidemic, 2011: Warning about the Dangers of Tobacco.

[2] Mathers C.D., Loncar D. (2006). Projections of global mortality and burden of disease from 2002 to 2030. PLOS Med..

[3] Office on Smoking and Health (US) (2006). The Health Consequences of Involuntary Exposure to Tobacco Smoke: A Report of the Surgeon General.

[4] Menezes A.M., Hallal P.C. (2007). Role of passive smoking on COPD risk in non-smokers. Lancet.

[5] Zheng P., Li W., Chapman S., Zhang Z., Gao J., Fu H. (2011). Workplace exposure to secondhand smoke and its association with respiratory symptoms—A cross-sectional study among workers in Shanghai. Tob. Control.

[6] Meeker J.D., Benedict M.D. (2013). Infertility, Pregnancy Loss and Adverse Birth Outcomes in Relation to Maternal Secondhand Tobacco Smoke Exposure. Curr. Womens Health Rev..

[7] Hyland A., Piazza K.M., Hovey K.M., Ockene J.K., Andrews C.A., Rivard C., Wactawski-Wende J. (2014). Associations of lifetime active and passive smoking with spontaneous abortion, stillbirth and tubal ectopic pregnancy: A cross-sectional analysis of historical data from the Women’s Health Initiative. Tob. Control.

[8] Rubin D.H., Krasilnikoff P.A., Leventhal J.M., Weile B., Berget A. (1986). Effect of passive smoking on birth-weight. Lancet.

[9] Qiu J., He X., Cui H., Zhang C., Zhang H., Dang Y., Han X., Chen Y., Tang Z., Zhang H. (2014). Passive smoking and preterm birth in urban China. Am. J. Epidemiol..

[10] Jaddoe V.W., Troe E.J., Hofman A., Mackenbach J.P., Moll H.A., Steegers E.A., Witteman J.C. (2008). Active and passive maternal smoking during pregnancy and the risks of low birthweight and preterm birth: The Generation R Study. Paediatr. Perinat. Epidemiol..

[11] Fantuzzi G., Aggazzotti G., Righi E., Facchinetti F., Bertucci E., Kanitz S., Barbone F., Sansebastiano G., Battaglia M.A., Leoni V. (2007). Preterm delivery and exposure to active and passive smoking during pregnancy: A case-control study from Italy. Paediatr. Perinat. Epidemiol..

[12] Anderson H.R., Cook D.G. (1997). Passive smoking and sudden infant death syndrome: Review of the epidemiological evidence. Thorax.

[13] Strachan D.P., Cook D.G. (1997). Health effects of passive smoking 1: Parental smoking and lower respiratory illness in infancy and early childhood. Thorax.

[14] Cook D.G., Strachan D.P. (1997). Health effects of passive smoking. 3. Parental smoking and prevalence of respiratory symptoms and asthma in school age children. Thorax.

[15] Yang G.H., Ma J.M., Liu N.A., Zhou L.N. (2005). Smoking and passive smoking in Chinese, 2002. Chin. J. Epidemiol..

[16] Chen M.H. (2007). Economic concerns hamper tobacco control in China. Lancet.

[17] Li Q., Hsia J., Yang G. (2011). Prevalence of Smoking in China in 2010. New Engl. J. Med..

[18] Gan Q., Smith K.R., Hammond S.K., Hu T. (2007). Disease burden of adult lung cancer and ischaemic heart disease from passive tobacco smoking in China. Tob. Control.

[19] Li Q., Xiao L., Zhao L., Morton J., Wang C., Feng G., Wu Y., Jiang Y., Yang J., Gan Q. Global Adult Tobacco Survey (GATS) China 2010 Country Report. http://www.notc.org.cn/newjcpg/201304/W020121108628365808856.pdf.

[20] Zhang L., Hsia J., Tu X., Xia Y., Zhang L., Bi Z., Liu H., Li X., Stanton B. (2015). Exposure to secondhand tobacco smoke and interventions among pregnant women in China: A systematic review. Prev. Chronic Dis..

[21] National Health and Family Planning Commission of China (2015). Report on China’s Migrant Population Development (2015).

[22] Mou J., Fellmeth G., Griffiths S., Dawes M., Cheng J. (2013). Tobacco smoking among migrant factory workers in Shenzhen, China. Nicotine Tob. Res..

[23] Chen X., Li X., Stanton B., Fang X., Lin D., Cole M., Liu H., Yang H. (2004). Cigarette smoking among rural-to-urban migrants in Beijing, China. Prev. Med..

[24] Yu Z., Qiao L., Wang X., Yang X., Zhang L., Hu W., Wu Y., Kong L., Du S., Ma G. (2010). Investigation on health-threatening behaviors among the immigrants living in an urban district of Beijing. Chin. J. Prev. Control Chronic Non-Commun. Dis..

[25] Zhu Z., Zhang M., Zhong S. (2013). Investigation of Health Related Factors of Migrant Workers Gathering Areas. Chin. Gen. Pract..

[26] Cui X., Rockett I.R., Yang T., Cao R. (2012). Work stress, life stress, and smoking among rural–urban migrant workers in China. BMC Public Health.

[27] Gong P., Liang S., Carlton E.J., Jiang Q., Wu J., Wang L., Remais J.V. (2012). Urbanisation and health in China. Lancet.

[28] Zhang J., Zhu Y., Fu Y., Ding D., Hou Z., Liu J. (2015). Investigation on the current situation of passive smoking among the floating population in Jilin province. Chin. J. Public Health Eng..

[29] Huang Z., Wang L., Zhang M., Deng Q., Wang Z., Zhao Y., Li Y., Zhao Z. (2014). Smoking behavior among the Chinese employed floating population aged 18–59 in 2012. Chin. J. Epidemiol..

[30] Department of Sociology at Sun Yat-sen University China Labor Force Dynamics Survey. http://css.sysu.edu.cn/Data.

[31] World Health Organization (WHO) (2014). Trends in Maternal Mortality: 1990 to 2013: Estimates by WHO, UNICEF, UNFPA, the World Bank and the United Nations Population Division.

[32] Ledwith F. (1983). Guidelines for the Conduct of Tobacco Smoking Surveys of the General Population.

[33] Fay M.P., Feuer E.J. (1997). Confidence intervals for directly standardized rates: A method based on the gamma distribution. Stat. Med..

[34] Selvin S. (2004). Statistical Analysis of Epidemiologic Data.

[35] Li Z., Yao Y., Yu Y., Shi J., Liu Y., Tao Y., Kou C., Zhang H., Han W., Yin Y. (2015). Prevalence and Associated Factors of Passive Smoking among Women in Jilin Province, China: A Cross-Sectional Study. Int. J. Environ. Res. Public Health.

[36] Yang T., Wu J., Rockett I., Abdullah A.S., Beard J., Ye J. (2009). Smoking patterns among Chinese rural–urban migrant workers. Public Health.

[37] Joseph L. (2012). Epidemic Models of the Onset of Social Activities. Handbook of Developmental Research Methods.

[38] Ennett S.T., Foshee V.A., Bauman K.E., Hussong A., Faris R., Hipp J.R., Cai L. (2010). A social contextual analysis of youth cigarette smoking development. Nicotine Tob. Res..

[39] Glad W., Adesso V.J. (1976). The relative importance of socially induced tension and behavioral contagion for smoking behavior. J. Abnorm. Psychol..

[40] Desalu O.O., Onyedum C.C., Adewole O.O., Fawibe A.E., Salami A.K. (2011). Secondhand smoke exposure among nonsmoking adults in two Nigerian cities. Ann. Afr. Med..

[41] King B.A., Mirza S.A., Babb S.D., GATS Collaborating Group (2013). A cross-country comparison of secondhand smoke exposure among adults: Findings from the Global Adult Tobacco Survey (GATS). Tob. Control.

[42] Protano C., Andreoli R., Manini P., Vitali M. (2012). How home-smoking habits affect children: A cross-sectional study using urinary cotinine measurement in Italy. Int. J. Public Health.

[43] Matt G.E., Quintana P.J., Destaillats H., Gundel L.A., Sleiman M., Singer B.C., Jacob P., Benowitz N., Winickoff J.P., Rehan V. (2011). Thirdhand tobacco smoke: Emerging evidence and arguments for a multidisciplinary research agenda. Environ. Health Perspect..

[44] Protano C., Vitali M. (2011). The new danger of thirdhand smoke: Why passive smoking does not stop at secondhand smoke. Environ. Health Perspect..

